# CD8+ T Cell Priming by Dendritic Cell Vaccines Requires Antigen Transfer to Endogenous Antigen Presenting Cells

**DOI:** 10.1371/journal.pone.0011144

**Published:** 2010-06-16

**Authors:** Alice W. Yewdall, Scott B. Drutman, Felecia Jinwala, Keith S. Bahjat, Nina Bhardwaj

**Affiliations:** 1 Cancer Institute, New York University School of Medicine, New York, New York, United States of America; 2 Earle A. Chiles Research Institute, Providence Cancer Center, Portland, Oregon, United States of America; New York University, United States of America

## Abstract

**Background:**

Immunotherapeutic strategies to stimulate anti-tumor immunity are promising approaches for cancer treatment. A major barrier to their success is the immunosuppressive microenvironment of tumors, which inhibits the functions of endogenous dendritic cells (DCs) that are necessary for the generation of anti-tumor CD8+ T cells. To overcome this problem, autologous DCs are generated *ex vivo*, loaded with tumor antigens, and activated in this non-suppressive environment before administration to patients. However, DC-based vaccines rarely induce tumor regression.

**Methodology/Principal Findings:**

We examined the fate and function of these DCs following their injection using murine models, in order to better understand their interaction with the host immune system. Contrary to previous assumptions, we show that DC vaccines have an insignificant role in directly priming CD8+ T cells, but instead function primarily as vehicles for transferring antigens to endogenous antigen presenting cells, which are responsible for the subsequent activation of T cells.

**Conclusions/Significance:**

This reliance on endogenous immune cells may explain the limited success of current DC vaccines to treat cancer and offers new insight into how these therapies can be improved. Future approaches should focus on creating DC vaccines that are more effective at directly priming T cells, or abrogating the tumor induced suppression of endogenous DCs.

## Introduction

Therapeutic vaccines are a considered a realistic approach for cancer immunotherapy, because animal studies have shown that recognition of tumor associated antigens (TAAs) by cytotoxic T lymphocytes can lead to the destruction of tumor cells [1], [2], [3]. In order to generate tumor-specific T cells with full effector function they first must undergo priming by dendritic cells (DCs), the antigen presenting cell (APC) most efficient at initiating potent CD8+ T-cell responses [4], [5]. Therefore, understanding how to modulate DC functions may be essential to therapeutically inducing anti-tumor immune responses [6], [7]. The immunomodulatory activity of DCs is regulated by the process of maturation. In response to environmental signals such as pathogens or inflammatory cytokines, immature DCs undergo phenotypic and qualitative changes to become mature DCs, characterized by presentation of peptides bound to major histocompatibility complexes (MHC), high surface expression of co-stimulatory molecules, secretion of cytokines, and potent T cell stimulatory abilities. Depending upon the maturation stimuli, DCs instruct T cells towards different types of immune responses [8], [9]. Thus, the development of mature DCs with the essential properties for induction of effective anti-tumor immunity is critical.

One significant barrier to therapeutic stimulation of anti-tumor immunity is constituents of the tumor microenvironment [10] such as secreted immunosuppressive factors [11], myeloid-derived suppressor cells [12], and regulatory T cells [13], that adversely affect DC activation and consequently blunt the generation of anti-tumor immunity. Furthermore, antigen presented to naïve T cells by DCs that were not properly activated can lead to T cell tolerance [14], [15], [16]. To circumvent this problem, many studies have focused on optimizing the antigen presentation function of DCs *ex vivo* such that these DCs can be adoptively transferred back into the *in vivo* environment. Ideally, this should yield DCs that, following injection, traffic to the draining lymph node and efficiently activate T cells, leading to the generation of an effective immune response [17], [18].

The optimization of *ex vivo* DC manipulation has focused primarily on maturation stimuli to enhance the expression of co-stimulatory molecules, secretion of pro-inflammatory cytokines, and expression of chemokine receptors important for migration to lymphoid organs. Additionally, the form of antigen used for loading DCs is a major consideration. The most commonly used form of antigens, are pre-determined immunogenic peptide epitopes derived from TAA, which are restricted to specific MHC haplotypes; or whole tumor antigens, which are theoretically more advantageous than peptide antigens because they can be processed into epitopes that can be presented regardless of patients' MHC haplotypes.

Several clinical trials conducted over the past decade have demonstrated that DC vaccines can prime and boost antigen-specific CD8+ T cells in humans. However, their clinical efficacy remains to be definitively demonstrated [6], [19], [20], [21]. The lack of success has been variously attributed to several factors: administration of relatively low cell numbers of DCs, suboptimal route of administration, improper antigen dose, poor choice of antigenic targets, unsuitable maturation state of DCs, and inappropriate frequency of injections. However, understanding exactly which of these concerns represent true problems may be difficult because little is known regarding the fate and function of *ex vivo* generated DCs after they have been injected. Because tracking these events in patients in a controlled manner is not feasible, we utilized a murine model of DC vaccination to better understand the events following DC injection. We show here that *ex vivo* derived DC vaccines have an insignificant role in the direct priming of T cells *in vivo*. Instead, evidence is provided that DC vaccines indirectly prime naïve CD8+ T cells *in vivo* by transferring antigens to endogenous cells, which subsequently present them to CD8+ T cells.

## Results

### CD8+ T cell priming by peptide loaded DC vaccine requires endogenous antigen presenting cells

**Figure 1:**
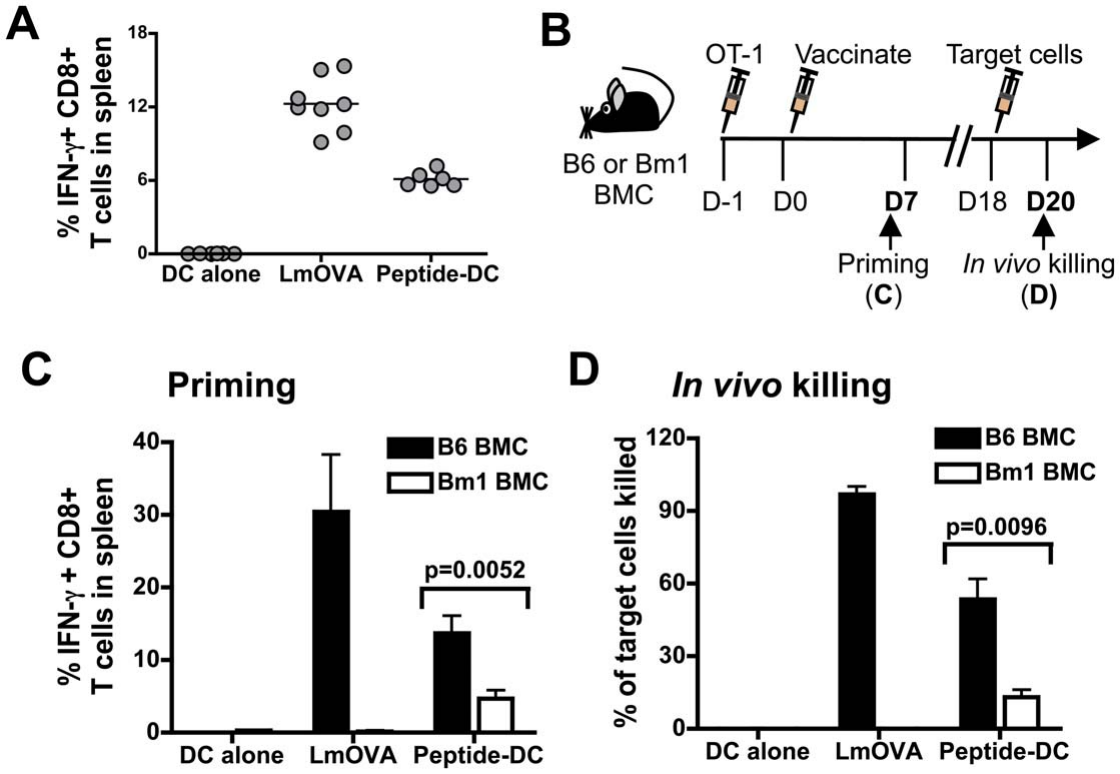
Peptide-pulsed DC vaccines require help from host immune cells to induce a maximal CD8+ T cell response. (***A***) Wild type C57BL/6 (B6) mice were vaccinated with either attenuated *Lm*OVA (1×10^6^ CFU/mouse), OVA_257–264_ (100 ng/ml)–pulsed LPS-matured B6 DCs (peptide-DCs, 1×10^6^ cells/mouse), or unpulsed LPS-matured B6 DCs (DC alone, 1×10^6^ cells/mouse). On day 7, the percentage of IFN-γ producing CD8+ T cells was determined (horizontal bar  =  mean). (***B***) B6→B6 (B6) and Bm1→B6 (Bm1) marrow chimeras (BMCs) were adoptively transferred with 5,000 naïve OT-1 T cells and vaccinated the following day with *Lm*OVA (1×10^6^ CFU/mouse), peptide-DCs (1×10^6^ cells/mouse), or DCs alone (1×10^6^ cells/mouse). The immune response was assessed on days 7 and 18 post-vaccination. (***C***) Antigen-specific IFN-γ production by splenic CD8+ T cells was measured on day 7. Data are mean + s.d. for 3 mice per group and are representative of 3 experiments. (***D***) *In vivo* killing of OVA_257–264_ peptide coated target cells in the spleen between days 18 and 20 was assessed. Specific killing was normalized to the internal control of target cells coated with an irrelevant peptide from the Sendai virus nucleoprotein, NP_324–332_. Data are mean ± s.d. for 3 mice per group and are representative of 2 experiments.

To investigate the immunogenicity of DC vaccines, we first established a murine model to monitor the *in vivo* activation of antigen-specific CD8+ T cells following vaccination. Mice were intravenously injected with *ex vivo* derived DCs pulsed with the MHC class I-restricted epitope of the ovalbumin (OVA) protein, OVA_257–264_ (peptide-DCs). As a control, mice were immunized with attenuated *Listeria monocytogenes* (*Lm*) expressing OVA (*Lm*OVA). Immunization of wild type C57BL/6 (B6) mice with either *Lm*OVA or peptide-DCs elicited an OVA_257–264_-specific immune response as demonstrated by antigen-specific IFN-γ production by CD8+ T cells (**Fig. 1** ***A***). While the T cell response to *Lm*OVA should depend on antigen presentation by the endogenous bone-marrow-derived hematopoietic cells [22], the priming by peptide-DCs in these mice could be a result of either direct interaction of the injected DCs with endogenous T cells, or antigens from the injected DCs captured by host cells and subsequently presented to T cells.

To examine the T cell priming that resulted from only the direct effect of the injected DCs, we repeated these vaccinations in bone marrow chimeric mice (BMCs) that lack a hematopoietic compartment able to present the OVA_257–264_ peptide. This was accomplished by reconstituting lethally irradiated wild type mice with bone marrow from Bm1 mice, which express a mutant H-2K^b^ allele (H-2K^bm1^) that prevent proper interaction of the OVA_257–264_-MHC complex with its cognate CD8+ T cell [23]. We confirmed donor-specific tolerance after allogeneic bone marrow transplantation (**Fig. S1**). Wild type and Bm1-derived bone marrow cells will reconstitute a T cell compartment with differing repertoires of naive antigen-specific T cells therefore, we normalized the number of OVA_257-264_-specific T cells by adoptively transferring 5,000 naive OT-I CD8+ T cells, into each mouse one day prior to vaccination (**Fig. 1** ***B***).

As expected, mice reconstituted with Bm1 bone marrow (Bm1 BMCs) did not elicit an OVA_257–264_-specific CD8+ T cell response after vaccination with *Lm*OVA, in contrast to mice reconstituted with wild type bone marrow (B6 BMCs) (**Fig. 1** ***C***). However, following intravenous vaccination with peptide-DCs, the number of IFN-γ producing CD8+ T cells generated was significantly reduced in Bm1 BMCs (**Fig. 1** ***C***), and this small number of T cells primed in the Bm1 BMCs also exhibited a reduced effector cell function (**Fig. 1** ***D***). These results suggest that the T cell priming observed after vaccination with peptide DCs in **Fig. 1** ***A*** requires a host with a hematopoietic compartment capable of capturing and re-presenting the vaccine derived antigen.

Antigen transfer from the injected DCs to endogenous APCs may occur either by shedding of peptides or transfer of intact peptide-MHC complexes [24], [25], possibly via exchange of plasma membranes and associated proteins between cells [26]. Contact-dependent transfer of peptide-MHC complexes from *ex vivo* derived DCs to splenic cell populations can occur with great efficiency *in vitro* (**Fig. S2**). However, the inefficient T cell activation in Bm1 BMCs suggests that translocation of peptide-MHC complexes to host APCs is unlikely the source responsible for T cell priming in B6 BMCs.

### Priming of CD8+ T cells by DCs loaded with protein antigen also requires endogenous antigen presenting cells

**Figure 2:**
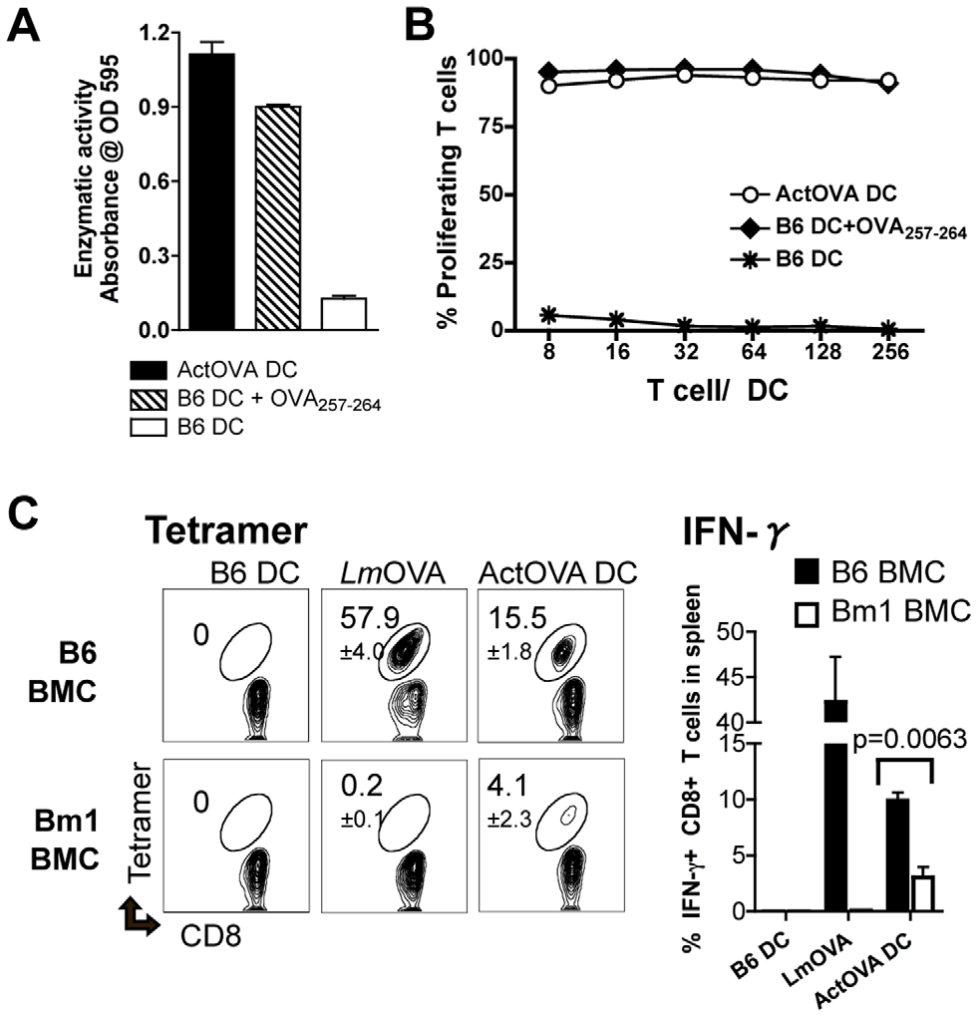
DC vaccines expressing protein antigens delivered intravenously require help from host cells for maximal activation of naïve CD8+ T cells. (***A***) By measuring the relative activation of B3Z T cell hybridomas, the cell surface OVA_257–264_-MHC complex levels on different LPS-matured bone marrow derived DC conditions were assessed. The DC conditions included LPS-matured B6 DCs, OVA_257–264_ (10 ng/ml)–pulsed LPS-matured B6 DCs, and ActOVA DCs. (***B***) To determine the ability of these DCs to activate naïve CD8+ T cells *in vitro*, they were cultured with CFSE-labeled naïve OT-1 T cells at various ratios. On day 3, the percentage of proliferating cells was analyzed by flow cytometry. (***C***) B6→B6 (B6) and Bm1→B6 (Bm1) bone marrow chimeras (BMCs) were adoptively transferred with 5,000 naïve OT-1 CD8+ T cells. The next day, these mice were vaccinated intravenously with one of the following: B6 DCs (0.5×10^6^ cells/mouse), attenuated *Lm*OVA (1×10^6^ CFU/mouse), or ActOVA DCs (0.5×10^6^ cells/mouse). The frequency of OVA_257–264_-tetramer positive and IFN-γ producing CD8+ T cells was determined 7 days post-vaccination (mean ± s.d. for 4 mice per group).

The heterogeneous nature of most tumor antigens and patient MHC haplotypes limits the practicality of externally-loaded pre-determined peptides as an antigen source for vaccines. Consequently, DCs are often loaded with antigens that require processing. To mimic this vaccine approach, we used DCs generated *ex vivo* from mice (Act-mOVA mice) [27] that constitutively express OVA protein under the actin promoter. DCs from Act-mOVA mice (ActOVA DCs) process the endogenous antigen in the MHC class I pathway and express OVA_257–264_-MHC class I complexes on their cell surface at a level comparable to the peptide-DCs used for vaccination (**Fig. 2** ***A***), and both types of DCs stimulate T cells *in vitro* with comparable potency (**Fig. 2** ***B***). When mice were intravenously immunized with ActOVA DCs using the same strategy described in **Fig. 1** ***B***, we again observed the same requirement for host APCs in generating a maximal CD8+ T cell response as we had with peptide-DCs (**Fig. 2** ***C***). Thus, regardless of whether the OVA antigen was originally expressed in DCs (ActOVA DCs) or externally loaded on the surface as a pre-determined epitope (peptide-DCs), maximal priming of OVA-specific T cells requires host APCs.

### The requirement for host antigen presenting cells does not depend on the route of DC vaccine delivery

**Figure 3:**
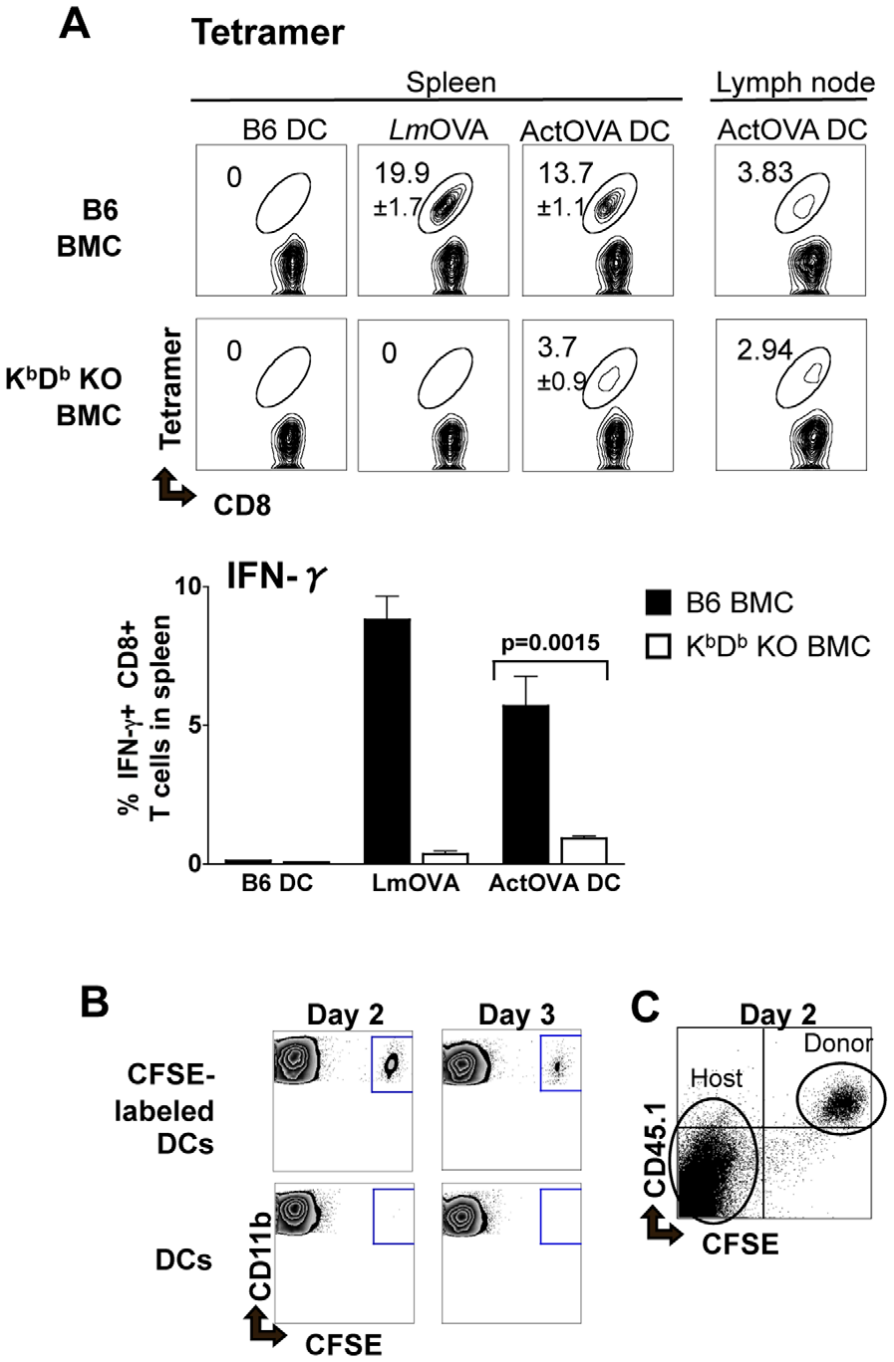
DCs vaccinated subcutaneously require MHC class I expression on the host immune cells to fully activate naïve CD8+ T cells. (***A***) B6→B6 (B6) and K^b^D^b^ KO→B6 (K^b^D^b^ KO) bone marrow chimeras (BMCs) were adoptively transferred with 5,000 naïve OT-1 CD8+ T cells. The next day, they were vaccinated subcutaneously in the footpad with one of the following: LPS-matured B6 DCs (0.5×10^6^ cells/mouse), attenuated *Lm-*OVA (1×10^6^ CFU/mouse), or LPS-matured ActOVA DCs (0.5×10^6^ cells/mouse). The frequencies of OVA_257–264_-tetramer positive CD8+ T cells in the spleen (individual mice) and draining lymph node (LN, combined value of from mice of each condition) and IFN-γ producing CD8+ T cells in the spleen of each mouse were determined 7 days post-vaccination (data are mean ± s.d. for 4 mice per group). (***B***) To track migration of DCs to the draining LN, CFSE-labeled or –unlabeled LPS-matured DCs (1×10^6^ cells/mouse) were injected subcutaneously in the foot pad of naïve mice. The popliteal LNs were removed 2 and 3 days post-injection. Cells were gated on the live (7-AAD negative) and CD11b+ population using flow cytometry to analyze the percentage (0.1–0.3%) of DCs that migrated. (***C***) CFSE-labeled CD45.1 donor DCs were injected subcutaneously in the footpad of CD45.1-CD45.2+ wild type mice. Two days later the popliteal LNs were removed and cells were stained with anti-CD45.1 and anti-CD45.2 antibodies.

Since the route of injection influences the sites DCs reach and thus may affect their function, we repeated our vaccination using subcutaneous injections. We also took this opportunity to extend our findings in an additional BMC system, in which irradiated wild type B6 mice were reconstituted with bone marrow from the MHC class I-deficient K^b^D^b^ KO mice (K^b^D^b^ KO BMCs) [28], [29]. Similar to our results with intravenously delivered DCs, transfer of antigens to host APCs was required for efficient CD8+ T cell priming (**Fig. 3** ***A***). The indirect role of the injected DCs in T cell priming was not due to a lack of exit from the injection site, because the DCs migrated to the draining lymph node (LN), albeit with a very low frequency of ≤0.5% of total injected cells (**Fig. 3** ***B***). This percentage of DCs to migrate is consistent with findings from other groups [30], [31], [32], [33]. Additionally, the DCs were viable and had not been internalized by host cells when detected in the draining LN two days after injection (**Fig. 3** ***C***). Our data demonstrate that, irrespective of the route of their delivery, optimal generation of systemic immune responses by DC vaccines requires host APCs.

### Differential requirements for endogenous DCs by peptide-loaded DCs versus protein expressing DCs

**Figure 4:**
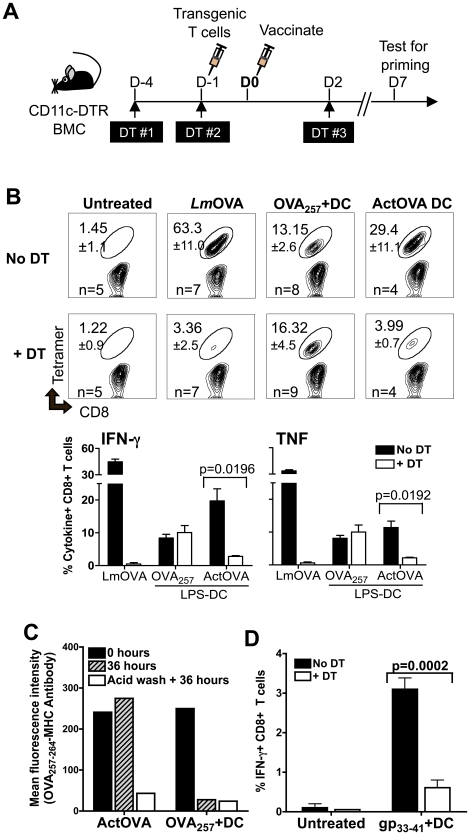
Endogenous CD11c^high^ DCs are recipients of antigens derived from DC vaccines, that are essential for activating naïve CD8+ T cells *in vivo.* (***A*** **-** ***B*** **,** ***D***) CD11c-DTR BMCs were treated with diptheria toxin (DT) on days −4, −1, and 2 relative to the day of vaccination. Naïve transgenic CD8+ T cells were adoptively transferred intravenously on day −1. Antigen-specific T cell responses were analyzed on day 7 post-vaccination. (***B***) Mice previously injected with naïve OT-1 CD8+ T cells (5,000 cells/mouse) were left untreated or vaccinated with one of the following: attenuated *Lm*OVA (1×10^6^ CFU/mouse), LPS-matured B6 DCs pulsed with 10 ng/ml of OVA_257–264_ peptide (OVA_257_+DCs, 0.5×10^6^ cells/mouse), or LPS-matured ActOVA DCs (0.5×10^6^ cells/mouse). The percentages of OVA_256–264_-tetramer positive and of IFN-γ- and TNF-producing CD8+ T cells are shown (data are mean ± s.d. for 4 to 9 mice per group as indicated). (***C***) ActOVA DC and peptide-DCs were cultured in the presence of proteosome inhibitors and the H-2K^b^-restricted NP_324–332_ peptides (1 ug/ml). Acid washing elutes surface MHC-bound peptides and was used to confirm the inhibition of antigen processing by the inhibitors. Shown are the cell surface levels of OVA_257–264_-MHC class I complexes. (***D***) DT-treated or untreated CD11c-DTR BMCs were injected with 10,000 LCMV P14 T cells, followed by vaccination with B6 bone marrow derived DCs pulsed with 1 ug/ml of LCMV gp_33–41_ peptides (peptide-DCs, 0.5×10^6^ cells/mouse). On Day 7 post-vaccination, the frequencies of IFN-γ producing CD8+ T cells (data are mean ± s.d. of 5 mouse per group) were determined.

Because DCs are the APC most efficient at priming CD8+ T-cells [6] it seemed likely that they might be the cell type responsible for the presentation of the DC vaccine-derived antigens we observed. To investigate this possibility, we repeated our vaccinations in irradiated wild type mice reconstituted with bone marrow from mice that express the primate diptheria toxin receptor (DTR) under control of the CD11c promoter (CD11c-DTR BMCs) [22] (**Fig. 4** ***A***). Treatment of these mice with diptheria toxin (DT) throughout the duration of the experiment selectively eliminated the CD11c^high^ expressing DCs as confirmed by flow cytometry (**Fig. S3**), and the lack of a T cell response in DT treated mice after infection with *Lm*OVA (**Fig. 4** ***B***).

Both peptide-DC and ActOVA DC vaccines primed a CD8+ T cell response efficiently in non-DT treated CD11c-DTR BMCs, as evidenced by the percentage of cells positive for OVA_257–264_-tetramer staining and antigen-specific IFNγ and TNF production by CD8+ T cells 7 days post-vaccination (**Fig. 4** ***B***). In DT-treated mice, vaccination with ActOVA DCs induced a poor T cell response, demonstrating that they rely substantially on host DCs to cross-prime antigens. However, the frequency of CD8+ T cells primed after delivery of peptide-DCs was not affected by the DT treatment.

In this model, T cell activation may reflect direct interaction of peptide-DCs with naive CD8+ T cells. However, in conjunction with previous results in Bm1 BMCs (**Fig. 1** ***C***), it is possible that peptide antigens from the injected peptide-DCs were transferred to cell types other than CD11c+ DCs, which subsequently activated naïve CD8+ T cells.

To determine how ActOVA DCs were unable to take advantage of peptide transfer to endogenous cells for T cell activation, we compared the stability of surface MHC-bound peptides on ActOVA DCs to their stability on peptide-DCs. We found that there is a greater dissociation rate of peptides that are externally loaded onto surface MHC class I molecules, than of peptides presented by ActOVA DCs, which are intracellularly processed and loaded (**Fig. 4** ***C***). Compared to ActOVA DCs, peptide-DCs seem to transfer their surface MHC-bound peptides more readily to host cells. We also confirmed that non-specific binding of peptides to non-MHC molecules on the peptide-DCs does not contribute to transfer of antigens to host cells resulting in T cell activation (**Fig. S4**).

The nature of antigen presentation by DCs is known to influence the quality of CD8+ T cells primed during an immune response [34]. The phenotypes of the OVA_257–264_-tetramer positive T cells primed by either type of DC vaccine, in the presence or absence of endogenous DCs were indistinguishable (**Fig. S5**). Because it seemed unlikely that non-CD11c+ DC populations would be able to efficiently prime a robust T cell response, we considered the possibility that the priming, which relied on endogenous hematopoetic cells, but was independent of endogenous DCs might be limited to very immunogenic peptides such as OVA_257–264_. When we used a peptide-pulsed DC vaccine loaded with a less immunogenic peptide, gp_33–41_ from the lymphocytic choriomeningitis virus (LCMV) glycoprotein, non-DC host cells in DT-treated mice were unable to generate the maximal CD8+ T cell response to this epitope (**Fig. 4** ***D***). T cell responses to tumor antigens are better represented in this scenario, and therefore our results indicate that endogenous DCs are important for the effectiveness of both peptide-pulsed and whole protein loaded DC vaccines.

## Discussion

Our finding that the effectiveness of murine DC vaccines relies considerably on endogenous APCs, has important implications for the use of DC vaccines in cancer immunotherapy. Currently, the efficacy of *ex vivo*-derived DC-based immunotherapy has still to be proven for human cancers [35], [36], [37], and the limited success has been attributed to a variety of factors regarding the preparation and administration of the vaccine, the disease stage of participants in experimental trials, or the heterogeneous nature of most tumors. The dependence on endogenous cells by antigen-loaded DCs could be a key underlying explanation for poor vaccine efficacy, especially considering that the clinical trial participants with late disease stage are likely to have an immunosuppressive tumor environment, rendering the endogenous cells ineffective.

We show that peptide-DCs and whole antigen-loaded DCs may have less substantial roles in directly activating naïve CD8+ T cells *in vivo*. Although OVA_257–264_-pulsed DCs were able to induce an immune response in the absence of endogenous CD11c+ DCs in DT-treated CD11c-DTR BMCs (**Fig. 4** ***B***), we hesitate to conclude that they were directly interacting with T cells *in vivo* for two reasons. First, DCs loaded with a less immunogenic peptide, gp_33–41_ were unable to induce an optimal antigen-specific CD8+ T cell response in mice lacking endogenous CD11c+ DCs (**Fig. 4** ***D***). Secondly, injection of OVA_257–264_-pulsed B6 DCs intravenously or subcutaneously into hosts that lack hematopoietic cells capable of presenting antigens resulted in a significant reduction of antigen-specific CD8+ T cells (**Fig. 1** ***C*** and **Fig. 3** ***A***).

Peptide-DCs carry antigens in the form of peptides already bound to their surface MHC molecules, therefore are unlikely to be used as an antigen source for cross presentation into the MHC class I antigen presentation pathway, if internalized by a recipient cell. It is most likely that peptides are being transferred directly onto the surface MHC class I molecules of host APCs for their subsequent presentation. In theory, any cell type expressing MHC class I molecules and co-stimulatory molecules could present the antigenic peptide donated from peptide-DCs to activate naïve T cells. Possibly, APCs not eliminated by DT treatment, such as plasmacytoid DCs [38], [39], [40] participated in the priming of T cells. However, we demonstrated with gp_33–41_ peptides that the dependence of peptide transfer from injected DCs to non-CD11c+ DC endogenous cell populations does not apply for all antigens.

Similar to peptide-DCs, priming of CD8+ T cells by ActOVA DCs was inefficient in mice with endogenous cells lacking the appropriate MHC molecules to present the OVA_257–264_ peptide (**Fig. 2** ***C*** and **Fig. 3** ***A***). In the CD11c-DTR model, the OVA antigen expressed by ActOVA DCs is cross-presented specifically by endogenous DCs. This data is consistent with previous reports that DCs are required for the cross-priming of cell-associated antigens [22]. Protein antigens can be transferred and processed for MHC class I presentation by host DCs following phagocytosis of apoptotic [41] or necrotic [42], [43] ActOVA DCs. Although these results are expected, we were surprised that there was a difference in the requirement of endogenous CD11c+ DCs for T cell activation by ActOVA DCs versus OVA_257–264_-pulsed DCs. An *in vitro* assay confirmed that peptides loaded externally onto cell surface bound MHC I molecules (peptide-DCs) are less stable than those internally processed on loaded onto MHC I molecules, as in ActOVA DCs (**Fig. 4** ***C***). We conclude that because ActOVA DCs may not shed peptides as readily as OVA_257–264_-pulsed DCs *in vivo*, they are dependent on cross-presentation by host DCs.

Under priming conditions where antigen transfer by cross-presentation or by peptide transfer to MHC class I molecules was not permitted, there was some residual T cell expansion. The specific interaction of injected *ex vivo* derived DCs and antigen-specific T cells has been visualized in the draining LN of mice [17], therefore it is possible that the small amount of T-cell activation may have resulted from direct priming by injected DCs. Alternatively, several groups have described the transfer of peptide-MHC complexes to recipient cells from either secreted exosomes [44], [45], [46], live cells via the acquisition of plasma membranes [26], or dead cells in a process called “cross-dressing” [24], [25]. *In vitro*, the transfer of peptide-MHC complexes from *ex vivo* derived DCs to splenic cell populations takes place efficiently (**Fig. S2**). However, we conclude that this mechanism is not as effective *in vivo*, because the expression of an appropriate MHC haplotype on host cells is essential for a proper T cell response. More importantly, we showed that the residual T cells primed in Bm1 BMCs after administering peptide-DCs were not able to effectively kill target cells (**Fig. 1** ***D***). In this setting, the initial number of antigen-specific CD8+ T cells produced early during immune activation seems to be important for the generation of a memory response. Altogether our data show that DC vaccines overcome their inability to directly prime T cells *in vivo* by transferring antigens to hematopoietic cells for subsequent antigen presentation.

In order for endogenous DCs to properly prime naïve CD8+ T cells, they must undergo maturation. Therefore, in addition to acting as an antigen source, the injected DCs may also cause activation of endogenous recipient DCs without further stimulation. It has been shown that cross-presentation of OVA-loaded irradiated Bm1 or B6 splenocytes by host APCs results in proliferation of antigen-specific T cells, even in the absence of additional adjuvants [47], [48]. In these circumstances, the activation of host DCs required for efficient cross-presentation may be explained by the potential release of TLR ligands during donor cell death [49]. In our studies, it is possible that the eventual death of the injected LPS-treated DC vaccines in this study may have contributed to the successful T cell activation in mice with a wild type hematopoietic compartment. Phagocytosis of LPS-treated apoptotic cells has recently been shown to mature DCs in a TLR-4 dependent manner [50]. Indeed, we showed that injection of mice with DCs coated with LPS caused an upregulation of maturation markers on a small number of endogenous splenic DCs (**Fig. S6**), pointing to a mechanism by which host APCs can undergo activation following exposure to adoptively administered DCs.

However, the immunosuppressive nature of most cancers is unfavorable to the proper activation of host DCs [10] and is therefore an obstacle for any vaccine that relies on these cells to initiate a T cell response. We speculate that this is one explanation for the poor efficacy of DC vaccines to eliminate tumors in clinical trials [20]. In this context, the adjuvant activity of the administered *ex vivo*-derived DCs may be inadequate for proper naïve T cell stimulation, and the benefits of antigen transfer could go undetected, unless combined with other immunotherapeutic strategies that focus on inhibiting the factors that contribute to the immunosuppressive tumor environment [20]. Future anti-cancer immunotherapeutics might also benefit from activating and targeting antigens to endogenous DCs directly [51].

## Materials and Methods

### Mice, bacteria strains, and cells

C57BL/6, Balb/c, and OT-1 Rag KO mice were purchased from Taconic. CD11c-DTR, Act-mOVA, K^b^D^b^ KO, and Bm1 mice were purchased from Jackson Laboratory. Mice were 5 weeks of age at the start of each experiment. All experimental procedures involving mice were performed with the approval of the New York University School of Medicine Committee on Animal Research under the protocol number 070407-02. Attenuated *Listeria monocytogenes* (*Lm*) expressing OVA (*Lm*OVA) and the B3Z T cell hybridomas were provided by Anza Therapeutics. B3Z assays were performed as previously described [52]. Mouse bone marrow-derived DCs were generated as described previously [53]. Briefly, 2×10^6^ bone marrow cells were cultured in media supplemented with 20ng/ml recombinant murine GM-CSF (Invitrogen), and differentiated DCs were used for experiments between days 8 and 9. To prepare mature DCs for vaccination, they were cultured with lipopolysaccharide (LPS, 100 ng/ml, Sigma Aldrich) for 15 hours. Peptide-pulsed DCs were prepared by incubating LPS-matured DCs with 100 ng/ml of peptides for 1 hour at 4°C.

### Peptides and flow cytometry reagents

The peptides H-2K^b^-restricted OVA_257–264_ (SIINFEKL) and H-2D^b^-restricted lymphocytic choriomeningitis virus (LCMV)-derived gp_33–41_ (KAVYNFATM, natural epitope), and their irrelevant peptide controls, H-2K^b^-restricted sendai virus-derived NP_324–332_ (FAPGNYPAL) and H-2D^b^-restricted influenza A-derived NP_366–374_ (ASNENMETM) were purchased from Sigma Aldrich. OVA_257–264_-specific tetramers were generated by the Vaccine and Cell Therapy facility at New York University School of Medicine. The antibodies specific for CD3 (clone 145-2C11), CD8 (clone 53.6.7), CD11c (clone N418), CD11b (clone M1/70), CD62L (clone MEL-14), CCR7 (clone 4B12), IL-2Rβ (clone 5H4), CXCR3 (clone CXCR3-173), CD45.1 (clone A20), CD45.2 (clone 104), IL-2 (clone JES6-5H4), IFN-γ (clone XMG1.2), and TNF (clone MP6-XT22) were purchased from Biolegend. Antibodies specific for KLRG-1 (clone 2F1), Bcl-2 (clone 10C4), and OVA_257–264_-MHC complex (clone eBio25-D1.16) were purchased from eBioscience. Flow cytometry was performed using the FACS Calibur (BD Biosciences). Analysis was done using FlowJo software (Tree Star).

### Peptide stability assay

Bone marrow derived DCs were incubated with the proteasome inhibitors epoxomicin (0.5 µM, Sigma Aldrich) and *clasto*-Lactacystin beta-lactone (1 µM, Sigma Aldrich) for the length of time indicated. To confirm inhibition of antigen presentation, acid washing on a portion of the DCs was done after 15 hours of culture. Briefly, cells were washed with PBS, incubated with 0.131 M sodium citrate and 0.066 M sodium phosphate pH 3.3 for 2 min at room temperature. The cells were washed 3 times and cultured in the presence of inhibitors again.

### Generation of bone marrow chimeras (BMCs)

Host mice were supralethally irradiated with a split dose of 1300 rad delivered 3 hours apart using a cesium source (Gammacell 40) at a dose rate of 95 rad/min. Following irradiation the mice were reconstituted with 2×10^6^ bone marrow cells that were depleted of T cells using anti-CD90 (Thy1.2) (Miltenyi Biotec) magnetic bead separation. For 2 weeks following irradiation, the BMCs were maintained on aqueous antibiotics by supplementing their drinking water with 0.4% Trimethoprim Sulfa (Sigma), which was changed every 3 days. The mice were used 8–12 weeks after engrafting.

### *In vivo* killing assay

The generation of an adaptive immune response by vaccination was analyzed by testing the ability of the immune system to kill target cells *in vivo* in an antigen-specific manner. To prepare the target cells, splenocytes from naïve C57BL/6 mice were divided into 2 groups, and incubated with 1 µg/ml of either OVA_257–264_ peptides or an irrelevant peptide for 1 hour at 37°C in serum-free RPMI. Next, the cells were washed and each group was stained with a different concentration (5 µM and 1.25 µM) of carboxyfluorescein succinimidyl ester (CFSE, Invitrogen) in PBS for 10 minutes. After extensive washing the cells were mixed at equal proportions and injected intravenously at 5.0×10^6^ cells/mouse into the previously vaccinated mice. After the number of days indicated, *in vivo* killing of the target cells in the spleen of each mouse was evaluated using flow cytometry.

### OT-1 CD8+ T cell proliferation assay

Naïve CD8+ T cells from OT-1 Rag-/- mice were isolated and stained with CFSE (5 µM). For the *in vitro* assay, these cells were co-cultured with different concentrations of APCs in a U-bottom 96-well plate. On day 3, the cells were stained with antibodies for the T cell markers, CD3 and CD8. 7-AAD was used to gate out dead cells. Flow cytometry was used to analyze CFSE dilution that occurs with each cell division.

### *Ex vivo* T cell stimulation and intracellular staining

Splenocytes from previously vaccinated mice were stimulated with peptide-pulsed CFSE-labeled naïve splenocytes from wild type mice in the presence of monensin (Biolegend) for 5 hours at 37°C. Next, cells were surface stained with antibodies against CD3 and CD8, followed by intracellular staining with antibodies against either IFN-γ or TNF. The final percentage of antigen-specific CD8+ T cell response was determined after gating out CFSE+ cells and subtracting the percentage of cytokine producing CD8+ T cells in the samples from each mouse that were stimulated with an irrelevant peptide.

### Statistical analysis

All statistical analysis was performed using a two-tailed unpaired Student's *t* test.

## Supporting Information

Figure S1 Generation of donor-specific tolerance by allogeneic bone marrow transplantation. CFSE (5µM)-labeled 5.0×10^6^ splenocytes from C57BL/6 (B6) mice were injected into MHC I-deficient (K^b^D^b^ KO) mice, and bone marrow chimeric mice (BMCs) reconstituted with either B6 (B6→B6 BMC) or K^b^D^b^ KO (K^b^D^b^ KO→B6 BMC) bone marrow cells. Seven days later, tolerance of the injected cells was measured by their presence detected on flow cytometry.
(4.30 MB TIF)

Figure S2 Peptide-MHC complexes are transferred to splenic cell populations efficiently in a contact-dependent manner *in vitro*. (A) To confirm that MHC class I-deficient cells cannot present antigens, splenocytes from wild type (B6) mice and from MHC class I-deficient K^b^D^b^ KO mice were pulsed with OVA257-264 peptides at 100 ng/ml. Next, CD11c+ DCs and Gr-1+ cells were sorted and co-cultured with CFSE-labeled naïve OT-1 CD8+ T cells. Proliferation of the T cells was analyzed 3 days later. (B) CD45.2+ splenocytes from either wild type B6 or K^b^D^b^ KO mice were co-cultured with 10 ng/ml OVA257-264-pulsed DCs generated from wild type mice (B6.SJL) with a differential congenic marker, CD45.1. The splenocytes and peptide-pulsed DCs were co-cultured either directly or separated by a transwell membrane overnight. Next, different CD45.2+ splenic cell populations (CD11c+ DCs, B220+ B cells, and Gr-1+ cells) were sorted and co-cultured with naïve OT-1 CD8+ T cells for 3 days, after which proliferation of the T cells were analyzed. This experiment was repeated twice.
(6.15 MB TIF)

Figure S3 Depletion of endogenous cDCs in CD11c-DTR bone marrow chimeras by diphteria toxin treatment. To deplete endogenous cDCs during vaccination, CD11c-DTR bone marrow chimeras (BMCs) were treated with diphteria toxin (DT) on days −4, −1, and 2 relative to vaccination days as shown in Fig. 4A. DC ablation in the spleen was confirmed in DT treated mice on days 0 and 2 by staining for CD11b+CD11c^high^+ cells.
(4.71 MB TIF)

Figure S4 Peptide transfer is not due to non-specific binding of peptides on *ex vivo* generated DCs. DCs generated from either C57BL/6 or K^b^D^b^ KO mice were pulsed with OVA257-264 peptides (1 ug/ml) for 1 hour at 4°C. After extensive washing, the cells were injected (0.5×10^6^ cells/mouse) into bone marrow chimeras generated by reconstituting lethally irradiated K^b^D^b^ KO mice with bone marrow from B6 mice (B6→ K^b^D^b^ KO BMCs). On day 6, antigen specific T cell responses were determined by measuring the percentage of OVA257-264-tetramer, IFN-γ, or IL-2 positive CD8+ T cells.
(4.63 MB TIF)

Figure S5 Stage specific phenotype of CD8+ T cells primed in the presence or absence of endogenous CD11c+ DCs is similar. Diptheria toxin (DT) treated or untreated CD11c-DTR bone marrow chimeras (BMCs) were vaccinated with either OVA257-264-pulsed DCs (peptide-DCs) or ActOVA DCs as in Fig. 4B. OVA257-264-tetramer positive CD8+ T cells in the spleens on day 7 were stained with specific markers (CD62L, KLRG-1, CCR7, CXCR3, Bcl-2, and IL-2Rβ) to determine their effector or memory stage.
(9.53 MB TIF)

Figure S6 Splenic DCs upregulate maturation markers in mice injected with LPS-treated *in vivo* derived DCs. C57BL/6 mice were injected with either immature DC or LPS-DCs, or left untreated. Approximately 24 hours later, splenic CD11c^high^ DCs were analyzed for expression levels of CD86 and CD40.
(0.42 MB TIF)

## References

[1] Berendt MJ, North RJ, Kirstein DP (1978). The immunological basis of endotoxin-induced tumor regression. Requirement for T-cell-mediated immunity.. J Exp Med.

[2] Bui JD, Schreiber RD (2007). Cancer immunosurveillance, immunoediting and inflammation: independent or interdependent processes?. Curr Opin Immunol.

[3] Bhardwaj N (2007). Harnessing the immune system to treat cancer.. J Clin Invest.

[4] Steinman RM, Witmer MD (1978). Lymphoid dendritic cells are potent stimulators of the primary mixed leukocyte reaction in mice.. Proc Natl Acad Sci U S A.

[5] Banchereau J, Steinman RM (1998). Dendritic cells and the control of immunity.. Nature.

[6] Steinman RM, Banchereau J (2007). Taking dendritic cells into medicine.. Nature.

[7] Steinman RM (2007). Dendritic cells: understanding immunogenicity.. Eur J Immunol.

[8] Munz C, Steinman RM, Fujii S (2005). Dendritic cell maturation by innate lymphocytes: coordinated stimulation of innate and adaptive immunity.. J Exp Med.

[9] Joffre O, Nolte MA, Sporri R, Reis e Sousa C (2009). Inflammatory signals in dendritic cell activation and the induction of adaptive immunity.. Immunol Rev.

[10] Rabinovich GA, Gabrilovich D, Sotomayor EM (2007). Immunosuppressive strategies that are mediated by tumor cells.. Annu Rev Immunol.

[11] Wang T, Niu G, Kortylewski M, Burdelya L, Shain K (2004). Regulation of the innate and adaptive immune responses by Stat-3 signaling in tumor cells.. Nat Med.

[12] Marigo I, Dolcetti L, Serafini P, Zanovello P, Bronte V (2008). Tumor-induced tolerance and immune suppression by myeloid derived suppressor cells.. Immunol Rev.

[13] Wing K, Onishi Y, Prieto-Martin P, Yamaguchi T, Miyara M (2008). CTLA-4 control over Foxp3+ regulatory T cell function.. Science.

[14] Hawiger D, Inaba K, Dorsett Y, Guo M, Mahnke K (2001). Dendritic cells induce peripheral T cell unresponsiveness under steady state conditions in vivo.. J Exp Med.

[15] Probst HC, McCoy K, Okazaki T, Honjo T, van den Broek M (2005). Resting dendritic cells induce peripheral CD8+ T cell tolerance through PD-1 and CTLA-4.. Nat Immunol.

[16] Luo X, Tarbell KV, Yang H, Pothoven K, Bailey SL (2007). Dendritic cells with TGF-beta1 differentiate naive CD4+CD25- T cells into islet-protective Foxp3+ regulatory T cells.. Proc Natl Acad Sci U S A.

[17] Stoll S, Delon J, Brotz TM, Germain RN (2002). Dynamic imaging of T cell-dendritic cell interactions in lymph nodes.. Science.

[18] Mempel TR, Henrickson SE, Von Andrian UH (2004). T-cell priming by dendritic cells in lymph nodes occurs in three distinct phases.. Nature.

[19] Lesterhuis WJ, Aarntzen EH, De Vries IJ, Schuurhuis DH, Figdor CG (2008). Dendritic cell vaccines in melanoma: from promise to proof?. Crit Rev Oncol Hematol.

[20] Melief CJ (2008). Cancer immunotherapy by dendritic cells.. Immunity.

[21] Schadendorf D, Ugurel S, Schuler-Thurner B, Nestle FO, Enk A (2006). Dacarbazine (DTIC) versus vaccination with autologous peptide-pulsed dendritic cells (DC) in first-line treatment of patients with metastatic melanoma: a randomized phase III trial of the DC study group of the DeCOG.. Ann Oncol.

[22] Jung S, Unutmaz D, Wong P, Sano G, De los Santos K (2002). In vivo depletion of CD11c(+) dendritic cells abrogates priming of CD8(+) T cells by exogenous cell-associated antigens.. Immunity.

[23] Nikolic-Zugic J, Carbone FR (1990). The effect of mutations in the MHC class I peptide binding groove on the cytotoxic T lymphocyte recognition of the Kb-restricted ovalbumin determinant.. Eur J Immunol.

[24] Dolan BP, Gibbs KD, Ostrand-Rosenberg S (2006). Dendritic cells cross-dressed with peptide MHC class I complexes prime CD8+ T cells.. J Immunol.

[25] Dolan BP, Gibbs KD, Ostrand-Rosenberg S (2006). Tumor-specific CD4+ T cells are activated by “cross-dressed” dendritic cells presenting peptide-MHC class II complexes acquired from cell-based cancer vaccines.. J Immunol.

[26] Harshyne LA, Watkins SC, Gambotto A, Barratt-Boyes SM (2001). Dendritic cells acquire antigens from live cells for cross-presentation to CTL.. J Immunol.

[27] Ehst BD, Ingulli E, Jenkins MK (2003). Development of a novel transgenic mouse for the study of interactions between CD4 and CD8 T cells during graft rejection.. Am J Transplant.

[28] Vugmeyster Y, Glas R, Perarnau B, Lemonnier FA, Eisen H (1998). Major histocompatibility complex (MHC) class I KbDb -/- deficient mice possess functional CD8+ T cells and natural killer cells.. Proc Natl Acad Sci U S A.

[29] Murali-Krishna K, Lau LL, Sambhara S, Lemonnier F, Altman J (1999). Persistence of memory CD8 T cells in MHC class I-deficient mice.. Science.

[30] Labeur MS, Roters B, Pers B, Mehling A, Luger TA (1999). Generation of tumor immunity by bone marrow-derived dendritic cells correlates with dendritic cell maturation stage.. J Immunol.

[31] Smith AL, Fazekas de St Groth B (1999). Antigen-pulsed CD8alpha+ dendritic cells generate an immune response after subcutaneous injection without homing to the draining lymph node.. J Exp Med.

[32] MartIn-Fontecha A, Sebastiani S, Hopken UE, Uguccioni M, Lipp M (2003). Regulation of dendritic cell migration to the draining lymph node: impact on T lymphocyte traffic and priming.. J Exp Med.

[33] Baumjohann D, Hess A, Budinsky L, Brune K, Schuler G (2006). In vivo magnetic resonance imaging of dendritic cell migration into the draining lymph nodes of mice.. Eur J Immunol.

[34] Pulendran B, Ahmed R (2006). Translating innate immunity into immunological memory: implications for vaccine development.. Cell.

[35] Soruri A, Zwirner J (2005). Dendritic cells: limited potential in immunotherapy.. Int J Biochem Cell Biol.

[36] Figdor CG, de Vries IJ, Lesterhuis WJ, Melief CJ (2004). Dendritic cell immunotherapy: mapping the way.. Nat Med.

[37] O'Neill DW, Adams S, Bhardwaj N (2004). Manipulating dendritic cell biology for the active immunotherapy of cancer.. Blood.

[38] Villadangos JA, Young L (2008). Antigen-presentation properties of plasmacytoid dendritic cells.. Immunity.

[39] Schlecht G, Garcia S, Escriou N, Freitas AA, Leclerc C (2004). Murine plasmacytoid dendritic cells induce effector/memory CD8+ T-cell responses in vivo after viral stimulation.. Blood.

[40] Salio M, Palmowski MJ, Atzberger A, Hermans IF, Cerundolo V (2004). CpG-matured murine plasmacytoid dendritic cells are capable of in vivo priming of functional CD8 T cell responses to endogenous but not exogenous antigens.. J Exp Med.

[41] Albert ML, Sauter B, Bhardwaj N (1998). Dendritic cells acquire antigen from apoptotic cells and induce class I-restricted CTLs.. Nature.

[42] Sauter B, Albert ML, Francisco L, Larsson M, Somersan S (2000). Consequences of cell death: exposure to necrotic tumor cells, but not primary tissue cells or apoptotic cells, induces the maturation of immunostimulatory dendritic cells.. J Exp Med.

[43] Sancho D, Joffre OP, Keller AM, Rogers NC, Martinez D (2009). Identification of a dendritic cell receptor that couples sensing of necrosis to immunity.. Nature.

[44] Raposo G, Nijman HW, Stoorvogel W, Liejendekker R, Harding CV (1996). B lymphocytes secrete antigen-presenting vesicles.. J Exp Med.

[45] Andre F, Chaput N, Schartz NE, Flament C, Aubert N (2004). Exosomes as potent cell-free peptide-based vaccine. I. Dendritic cell-derived exosomes transfer functional MHC class I/peptide complexes to dendritic cells.. J Immunol.

[46] Luketic L, Delanghe J, Sobol PT, Yang P, Frotten E (2007). Antigen presentation by exosomes released from peptide-pulsed dendritic cells is not suppressed by the presence of active CTL.. J Immunol.

[47] Carbone FR, Bevan MJ (1990). Class I-restricted processing and presentation of exogenous cell-associated antigen in vivo.. J Exp Med.

[48] Li M, Davey GM, Sutherland RM, Kurts C, Lew AM (2001). Cell-associated ovalbumin is cross-presented much more efficiently than soluble ovalbumin in vivo.. J Immunol.

[49] Apetoh L, Ghiringhelli F, Tesniere A, Obeid M, Ortiz C (2007). Toll-like receptor 4-dependent contribution of the immune system to anticancer chemotherapy and radiotherapy.. Nat Med.

[50] Blander JM, Medzhitov R (2006). Toll-dependent selection of microbial antigens for presentation by dendritic cells.. Nature.

[51] Bonifaz LC, Bonnyay DP, Charalambous A, Darguste DI, Fujii S (2004). In vivo targeting of antigens to maturing dendritic cells via the DEC-205 receptor improves T cell vaccination.. J Exp Med.

[52] Shastri N, Gonzalez F (1993). Endogenous generation and presentation of the ovalbumin peptide/Kb complex to T cells.. J Immunol.

[53] Lutz MB, Kukutsch N, Ogilvie AL, Rossner S, Koch F (1999). An advanced culture method for generating large quantities of highly pure dendritic cells from mouse bone marrow.. J Immunol Methods.

