# Supplementary figures and images for: CD8+ T Cell Priming by Dendritic Cell Vaccines Requires Antigen Transfer to Endogenous Antigen Presenting Cells
Source: PLoS One. 2010 Jun 16;5(6):e11144. doi: 10.1371/journal.pone.0011144 (PMC2886840; doi:10.1371/journal.pone.0011144)

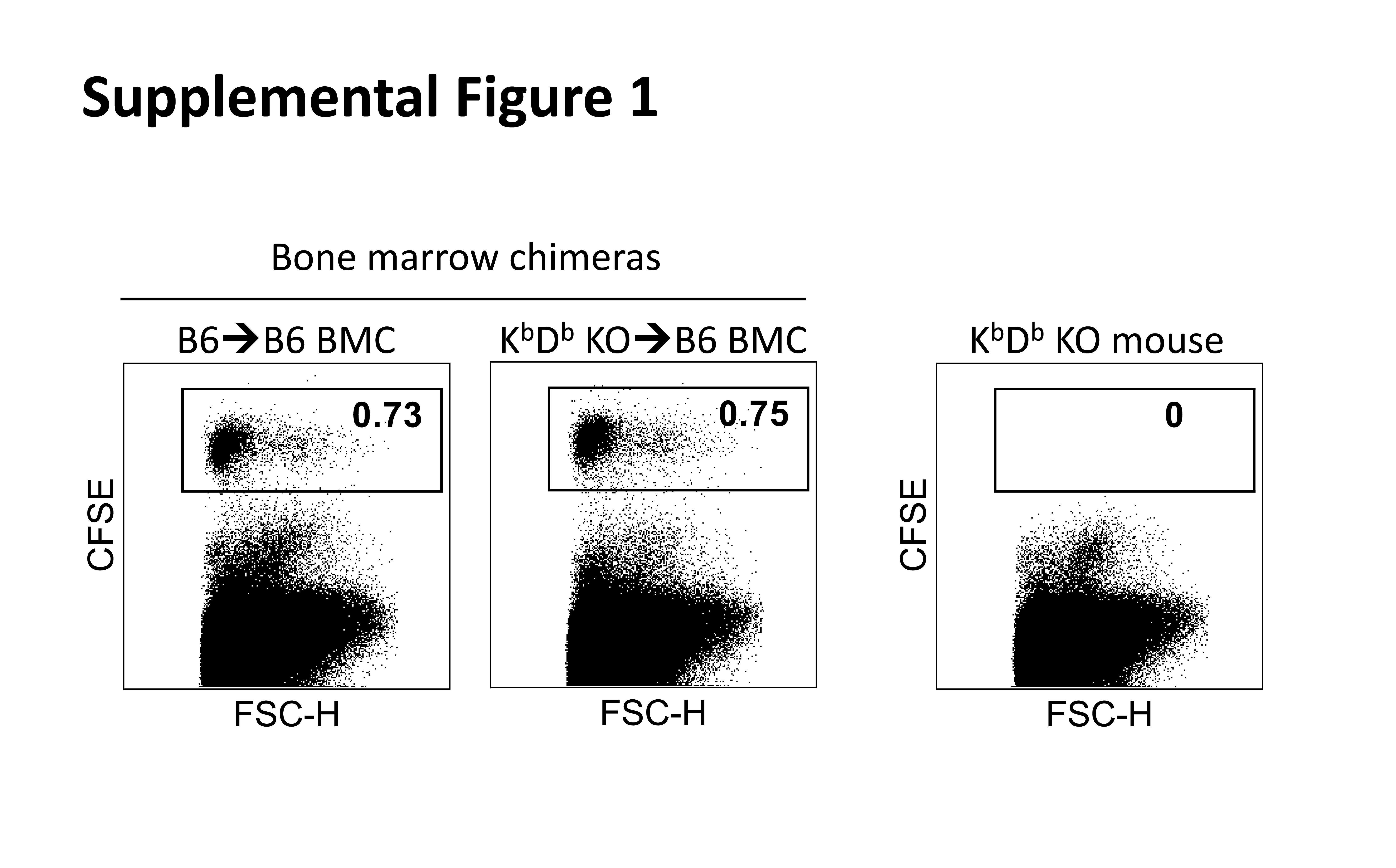

Supplement: Figure S1 — Generation of donor-specific tolerance by allogeneic bone marrow transplantation. CFSE (5µM)-labeled 5.0×10^6^ splenocytes from C57BL/6 (B6) mice were injected into MHC I-deficient (KbDb KO) mice, and bone marrow chimeric mice (BMCs) reconstituted with either B6 (B6→B6 BMC) or KbDb KO (KbDb KO→B6 BMC) bone marrow cells. Seven days later, tolerance of the injected cells was measured by their presence detected on flow cytometry. (4.30 MB TIF) [file pone.0011144.s001.tif]

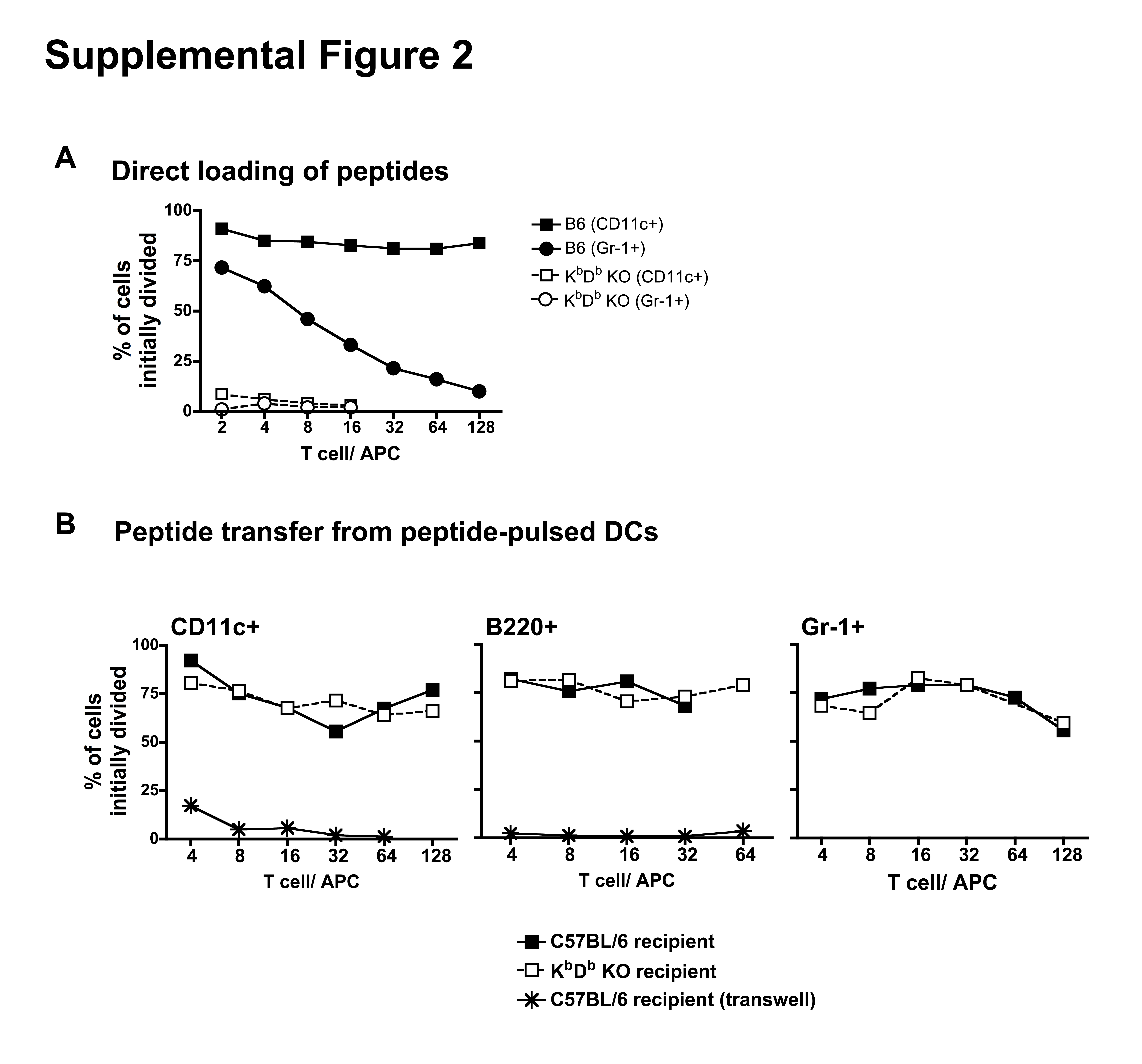

Supplement: Figure S2 — Peptide-MHC complexes are transferred to splenic cell populations efficiently in a contact-dependent manner in vitro. (A) To confirm that MHC class I-deficient cells cannot present antigens, splenocytes from wild type (B6) mice and from MHC class I-deficient KbDb KO mice were pulsed with OVA257-264 peptides at 100 ng/ml. Next, CD11c+ DCs and Gr-1+ cells were sorted and co-cultured with CFSE-labeled naïve OT-1 CD8+ T cells. Proliferation of the T cells was analyzed 3 days later. (B) CD45.2+ splenocytes from either wild type B6 or KbDb KO mice were co-cultured with 10 ng/ml OVA257-264-pulsed DCs generated from wild type mice (B6.SJL) with a differential congenic marker, CD45.1. The splenocytes and peptide-pulsed DCs were co-cultured either directly or separated by a transwell membrane overnight. Next, different CD45.2+ splenic cell populations (CD11c+ DCs, B220+ B cells, and Gr-1+ cells) were sorted and co-cultured with naïve OT-1 CD8+ T cells for 3 days, after which proliferation of the T cells were analyzed. This experiment was repeated twice. (6.15 MB TIF) [file pone.0011144.s002.tif]

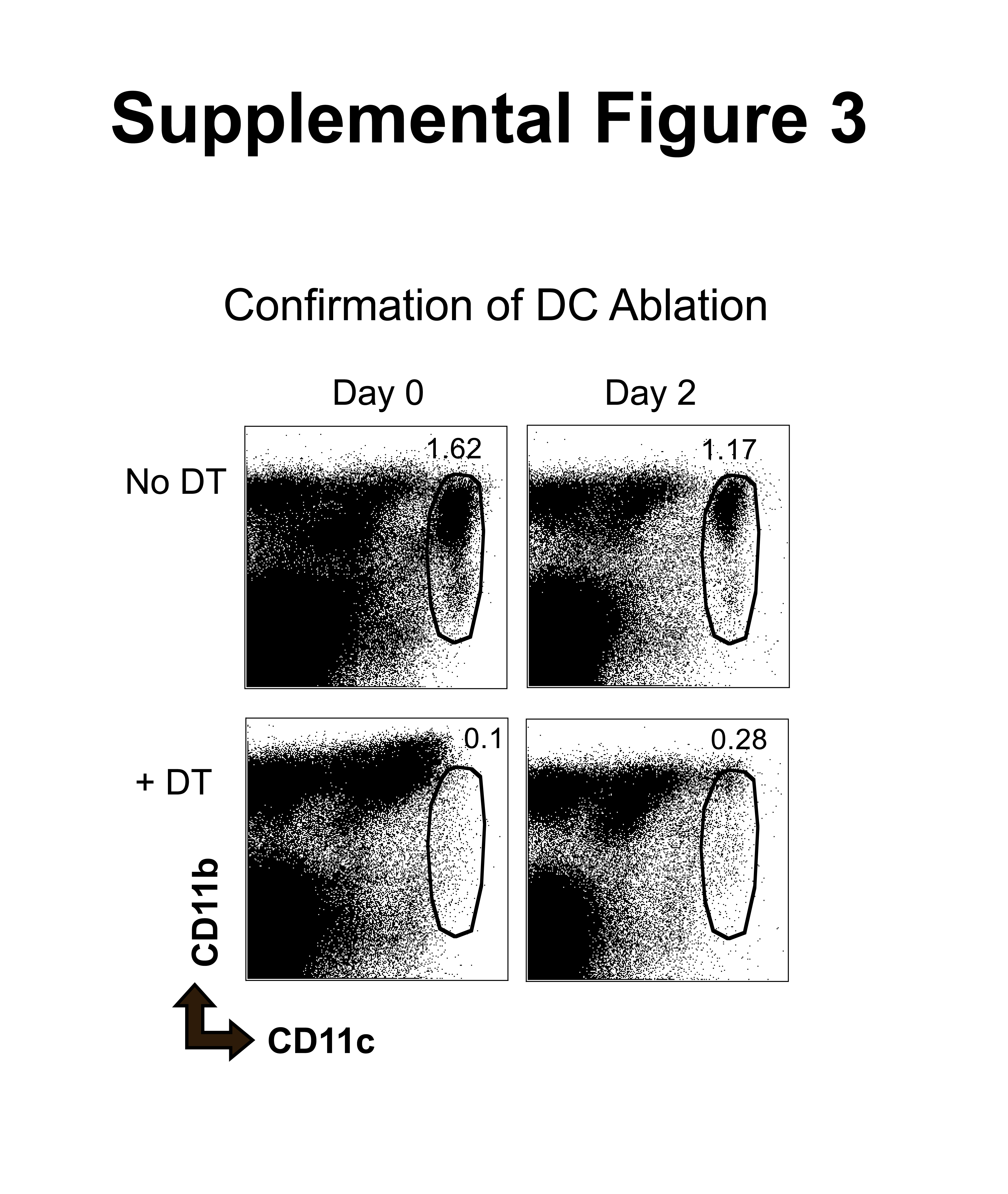

Supplement: Figure S3 — Depletion of endogenous cDCs in CD11c-DTR bone marrow chimeras by diphteria toxin treatment. To deplete endogenous cDCs during vaccination, CD11c-DTR bone marrow chimeras (BMCs) were treated with diphteria toxin (DT) on days −4, −1, and 2 relative to vaccination days as shown in Fig. 4A. DC ablation in the spleen was confirmed in DT treated mice on days 0 and 2 by staining for CD11b+CD11chigh+ cells. (4.71 MB TIF) [file pone.0011144.s003.tif]

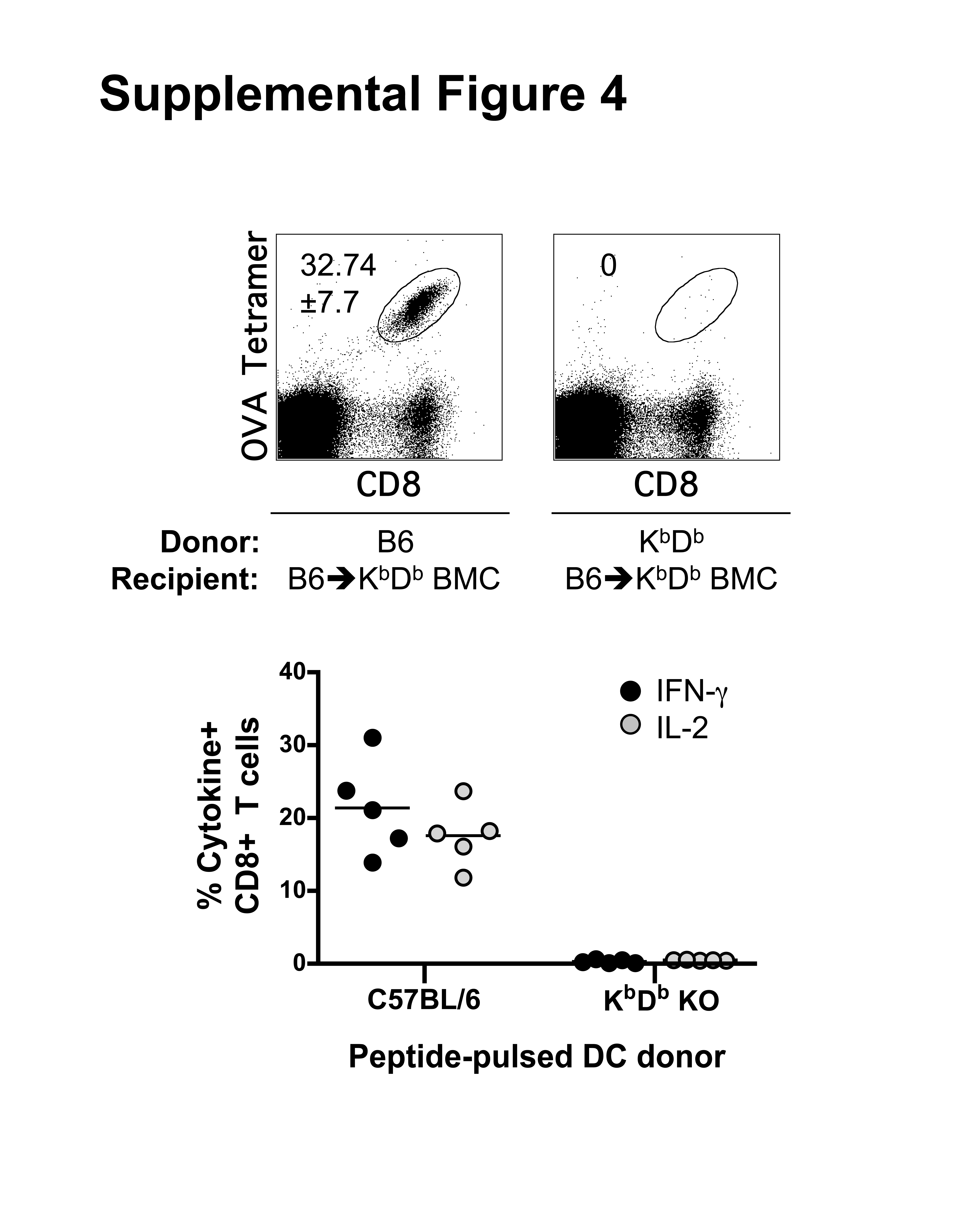

Supplement: Figure S4 — Peptide transfer is not due to non-specific binding of peptides on ex vivo generated DCs. DCs generated from either C57BL/6 or KbDb KO mice were pulsed with OVA257-264 peptides (1 ug/ml) for 1 hour at 4°C. After extensive washing, the cells were injected (0.5×10^6^ cells/mouse) into bone marrow chimeras generated by reconstituting lethally irradiated KbDb KO mice with bone marrow from B6 mice (B6→ KbDb KO BMCs). On day 6, antigen specific T cell responses were determined by measuring the percentage of OVA257-264-tetramer, IFN-γ, or IL-2 positive CD8+ T cells. (4.63 MB TIF) [file pone.0011144.s004.tif]

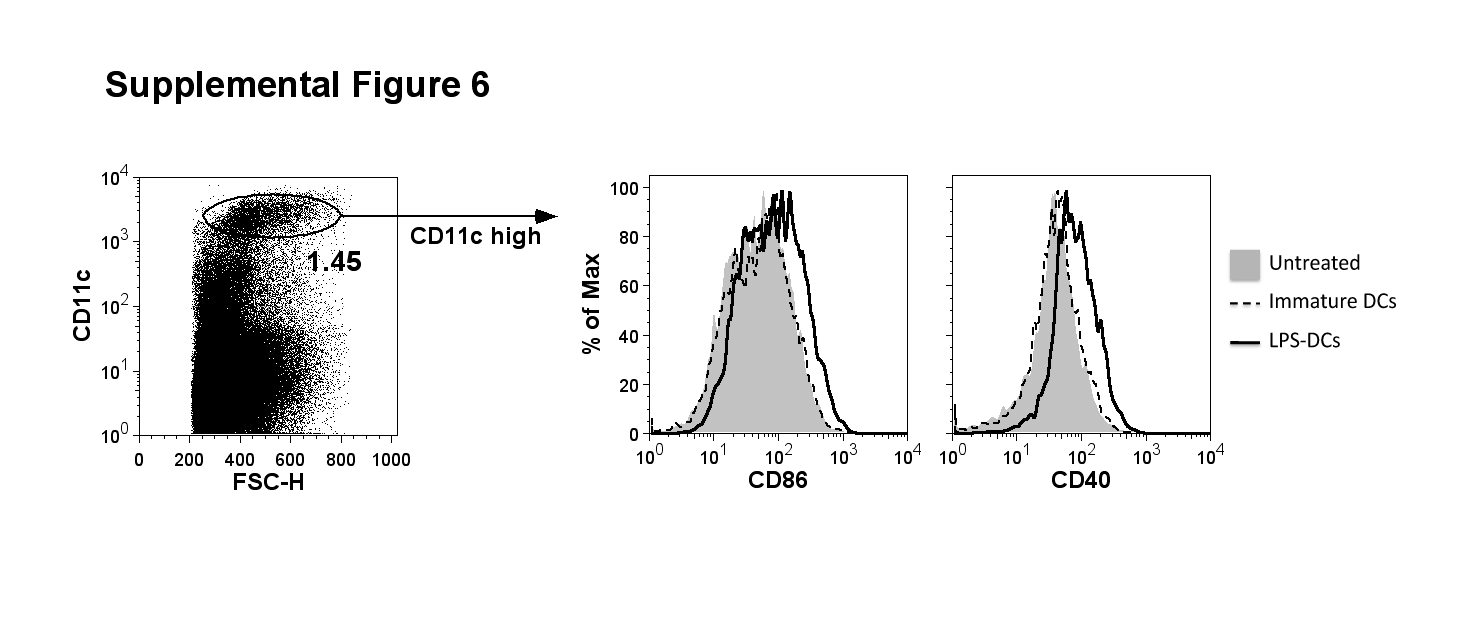

Supplement: Figure S6 — Splenic DCs upregulate maturation markers in mice injected with LPS-treated in vivo derived DCs. C57BL/6 mice were injected with either immature DC or LPS-DCs, or left untreated. Approximately 24 hours later, splenic CD11chigh DCs were analyzed for expression levels of CD86 and CD40. (0.42 MB TIF) [file pone.0011144.s006.tif]
